# An optimal brain tumor segmentation algorithm for clinical MRI dataset with low resolution and non-contiguous slices

**DOI:** 10.1186/s12880-022-00812-7

**Published:** 2022-05-14

**Authors:** Dheerendranath Battalapalli, B. V. V. S. N. Prabhakar Rao, P. Yogeeswari, C. Kesavadas, Venkateswaran Rajagopalan

**Affiliations:** 1 Department of Electrical and Electronics Engineering, Birla Institute of Technology and Science Pilani, Hyderabad Campus, Hyderabad, 500078 India; 2 Department of Pharmacy, Birla Institute of Technology and Science Pilani, Hyderabad Campus, Hyderabad, 500078 India; 3 Department of Imaging Sciences and Interventional Radiology, Sree Chitra Tirunal Institute for Medical Sciences and Technology, Trivandrum, 695011 India

**Keywords:** Brain tumor, Segmentation, Deep neural network, MRI

## Abstract

**Background:**

Segmenting brain tumor and its constituent regions from magnetic resonance images (MRI) is important for planning diagnosis and treatment. In clinical routine often an experienced radiologist delineates the tumor regions using multimodal MRI. But this manual segmentation is prone to poor reproducibility and is time consuming. Also, routine clinical scans are usually of low resolution. To overcome these limitations an automated and precise segmentation algorithm based on computer vision is needed.

**Methods:**

We investigated the performance of three widely used segmentation methods namely region growing, fuzzy C means and deep neural networks (deepmedic). We evaluated these algorithms on the BRATS 2018 dataset by choosing randomly 48 patients data (high grade, n = 24 and low grade, n = 24) and on our routine clinical MRI brain tumor dataset (high grade, n = 15 and low grade, n = 28). We measured their performance using dice similarity coefficient, Hausdorff distance and volume measures.

**Results:**

Region growing method performed very poorly when compared to fuzzy C means (fcm) and deepmedic network. Dice similarity coefficient scores for FCM and deepmedic algorithms were close to each other for BRATS and clinical dataset. The accuracy was below 70% for both these methods in general.

**Conclusion:**

Even though the deepmedic network showed very high accuracy in BRATS challenge for brain tumor segmentation, it has to be custom trained for the low resolution routine clinical scans. It also requires large training data to be used as a stand-alone algorithm for clinical applications. Nevertheless deepmedic may be a better algorithm for brain tumor segmentation when compared to region growing or FCM.

## Introduction

According to the World health organization (WHO) report nearly 0.3 million new brain tumor cases were diagnosed in 2018 across different age groups from preadolescence to adult [1], 2]. In general, brain tumors can be malignant (with cancerous symptoms) or benign (no cancerous symptoms) and. are distinguished by certain characteristics such as tumor growth rate, growth pattern, etc. [3], 4].

Glioma is the most common form of primary brain tumor with statistics [5], 6] showing an increase in glioma cases from 1973 to 2014. The brain tumors are comprehensively classified and graded using the diagnostic lexicon published by the WHO [6]. Gliomas are typically categorized into: low-grade glioma (LGG) and high-grade glioma (HGG). LGG are primarily considered as benign tumors with slow growth rate whereas, HGG, are cancerous tumors with rapid progression in the brain gradually leading to morbidity and mortality. Among the HGG tumors, glioblastomas are the most common type which affects nearly 55.6% of the people from all the age groups with the lowest prognosis rate and least survival rate [7]. According to the recent statistics [8] the chances of survival in HGG patients aged between 20–44 years and 55–65 years is 22% and 6%.

The current clinical standard procedure to establish diagnosis and decide therapeutic choices for brain tumor is biopsy [7], 9, 10]. Biopsy is an invasive procedure with potential complications like hematoma [11]. Therefore, a non-invasive neuroimaging-based radiological biomarker to delineate LGG and HGG tumors can immensely reduce cost, time and potential complications of surgery. The first step towards identifying neuroimaging-based biomarker requires segmentation of the brain tumor and its components from the surrounding healthy tissue. In addition to tumor segmentation delineating the different components of the tumor namely necrotic region, active region and edema surrounding the tumor is of vital importance. It provides crucial information about the nature of disease progression, treatment planning and the patient’s response to a therapeutic paradigm [12]. Manual brain tumor segmentation is done by an experienced radiologist using radiographic images routinely. This is prone to operator bias, has poor reproducibility [13] and is very time consuming; therefore, an automated reproducible segmentation method/algorithm is required. However, due to the heterogeneous nature of the tumor shape, size and frequency of its occurrence in different brain anatomical regions, automatic tumor segmentation still remains an open-ended challenge despite the continuous rigorous research in this field for more than two decades [14], 8]. Among the different neuroimaging modalities such as computed tomography, positron emission tomography and magnetic resonance imaging (MRI), MRI is the commonly used neuroimaging modality for diagnosis and treatment of brain tumors. This is due to the fact that MRI has high contrast to noise ratio, does not involve any ionizing radiation and can provide multi-modal 3D image sequences with versatile tissue contrast for better visualization. Among different MR sequences used in radiological diagnosis of brain tumor the most common ones include T1-weighted (T1-w), contrast (Gadolinium) enhanced T1-weighted (T1-Gd), T2-weighted (T2-w) and fluid attenuation by inversion recovery (FLAIR) sequences [15–17]. Nevertheless, the above mentioned qualitative MR sequences fails to distinguish (a) tumor recurrence from old tumors [7], (b) tumors from non-tumoral lesions such as ischemia, and (c) between different tumor grades [12]. Hence, an automatic brain tumor segmentation method is required to separate the tumor and its components (edema, necrotic region and active region) from its surrounding healthy tissue.

Active research in brain tumor segmentation field has led to a plethora of methods for brain tumor segmentation ranging from simple image thresholding to the latest deep neural networks [18]. Different brain tumor segmentation algorithms proposed using MRI, have their own limitations for e.g., variation in the tumor segmentation accuracy, scanner type, etc. which makes it difficult to find a stand-alone algorithm for the tumor segmentation. Our previous algorithm [27] based on region growing approach using brain symmetry to segment the brain tumor works well on high resolution 3D brain tumor images from BRATS dataset, but gave poor accuracy when brain tumor is located asymmetrically in the brain hemispheres and for clinical dataset which are usually 2D acquisitions with non-contiguous image slices. The tumor segmentation methods were classified hierarchically based on its complexity [19] and the region growing method ranks low. Fuzzy-C-means (FCM) clustering algorithm which occupies middle position in the hierarchy has shown promising results in segmenting asymmetrical brain tumors [20] for which region growing algorithm failed. However, the conventional FCM is prone to give incongruous segmentation when MRI has ringing artefacts, noise and intensity inhomogeneity thereby, misclassifying the tumors. Therefore, the overall performance of FCM is not on par with the current advanced neural networks based algorithms [21]. Neural network algorithms especially deep neural networks (DNN) have produced robust, highly accurate segmentation results in different medical imaging applications [22]. Therefore, there is a great interest in using DNN for, tumor classification, and for patient’s survival rate prediction [23].

DNN based tumor segmentation research has used BRATS MR image database for training and testing the performance of their network [18]. Although these methods were successful in segmenting tumors, their accuracy varies [24]. Moreover, since these algorithms were developed based on BRATS database their performance on routine clinical MRI data (which may have low resolution and non-contiguous slices) is not known. Since the routine clinical MR image acquisition protocol varies from site to site they differ from BRATS database for e.g. image resolution, inter slice gaps, etc.). Hence, in this study we aimed compare and contrast different brain tumor segmentation algorithms (from low to high end in the hierarchy) i.e. the region growing, FCM and DNN using our clinical dataset as well as BRATS dataset. By doing this we aimed to identify a robust automated brain tumor segmentation algorithm that suits best for the clinical data like ours.

## Methods

### Data acquisition

For this study 15 HGG and 28 LGG patients data were acquired at Sree Chitra Tirunal Institute of Medical Science and Technology hospital (SCTIMST) (Thiruvananthapuram, India). Institutional Ethics committee (IEC Regn No. ECR/189/Inst/KL/2013/RR-16) at Sree Chitra Tirunal Institute of Medical Science and Technology, Thiruvananthapuram, India approved this study waiving patient informed consent as this is a retrospective study. The approval number is IEC/1177. All procedures were performed in accordance with relevant guidelines.

From the BRATS 2018 dataset [18], 25, 26] we choose randomly 48 patients data (HGG, n = 24 and LGG, n = 24). This include T1w-gadolinium contrast enhanced image and FLAIR image sequences of each patient. 3D T1w-gadolinium contrast enhanced image and FLAIR images have an in plane resolution of 1 × 1 mm^2^, slice thickness = 1 mm, and the image matrix dimension of 512 × 464 × 160.

### Imaging protocol our clinical data

In SCTIMST patients were scanned using 1.5T Siemens MRI scanner (Magnetom Avanto, Erlangen, Germany). The MR sequences include: (1) 2D T2-w images acquired with repetition time (TR) = 5860 ms, time of echo (TE) = 110 ms; (2) FLAIR images were acquired with repetition time (TR) = 9000 ms, inversion time (TI) = 2500 ms, time of echo (TE) = 89 ms; (3) 2D T1w images were acquired with repetition time (TR) = 468 ms, time of echo (TE) = 11 ms and (4) 3D gradient echo was used to acquire T1w-gadolinum contrast enhanced images whose imaging parameters include repetition time (TR) = 9 ms, time of echo (TE) = 3.34 ms. The spatial dimensions of 2D-T2-w, FLAIR, T1-w and T1w-gadolinum contrast enhanced images include: (a) for T2-w and FLAIR images in plane resolution = 512 × 448, slice thickness = 5 mm, inter slice gap = 6.5 mm, for T1-w, in plane resolution = 320 × 270, slice thickness = 5 mm, inter slice gap = 6.5 mm and for T1-contrast enhanced images in plane resolution = 512 × 464, slice thickness = 0.9 mm, no slice gap.

### Data processing

Image pre-processing steps include (a) brain extraction using FSL BET tool (version 6.0.4). (b) Brain extracted images were then corrected for intensity inhomogeneity using FAST tool in FSL (version: v6.0, https://fsl.fmrib.ox.ac.uk/). For the BRATS 2018 brain tumor dataset we downloaded both the raw MR images and the segmented ground truth tumor images. The tumor regions from our clinical MRI images were segmented using ITK snap tool [39]. It was done under the supervision of an experienced radiologist (one of the co-authors CK). The pre-processed images were then given as input to the three different brain tumor segmentation algorithms namely a) region growing, (b) fuzzy C-means and (c) deep convolutional neural network. The performance of these three algorithms were evaluated using BRATS 2018 data and on our clinical data.

### Region growing method

The region growing (RG) algorithm developed in our previous study [27] was used for brain tumor segmentation. The algorithm was based on the following assumptions: (1) tumor is present either in the left hemisphere or in right hemisphere but not in both (2) left and right brain hemispheres exhibits symmetry. The algorithm consists of the following image processing steps (a) skull tripping and bias correction, (b) geometrical transformation, (c) separation of left and right brain hemispheres and (d) contrast stretching and region growing operation to segment the brain tumor. Steps (b)–(d) were implemented using MATLAB (2018b version).The pipeline comprises of: (a) bias corrected 3D MRI images were read into MATLAB using ‘niftiread’ command (b) geometrical transformation: input images were aligned vertically in such a way that the two hemispheres can be separated equally into two halves (c) the geometrically transformed MRI slices were separated into left and right hemisphere image volumes by subtracting each voxel in the right and left hemisphere images with the mirrored left and right hemisphere images to locate the tumor region. (d) After locating the tumor in either of the hemispheres, contrast stretching operation was performed to enhance the voxel intensity levels in the tumor region and to eliminate the surrounding unwanted voxels from the image slices. (e) subsequently the custom developed region growing algorithm was applied on the images containing tumor to group all the connected voxels with similar intensity levels to extract the whole tumor region (edema + core tumor region). The performance of this region growing algorithm was evaluated using the BRATS 2018 dataset and our clinical dataset. The efficacy of this segmentation algorithm was assessed by comparing the segmented output from this algorithm with the ground truth. Dice similarity coefficient values (DSC), volume of the tumor and its components and Hausdorff measurement were used as performance metrics. DSC is widely used in the literature [28] to analyse the accuracy of algorithms used in brain tumor segmentation. Hausdorff distance measurement was used to compare boundary voxels of the segmented regions between the ground truth and the results obtained from brain tumor segmentation algorithms [29], 30]. We also used the volume of the tumor and its components measure as performance metric as it is very often used clinically in the diagnosis and prognosis of brain tumors.

### Fuzzy C-means clustering (FCM) method

The second algorithm that we used to assess its performance on our clinical dataset was fuzzy C-means clustering method **(**FCM) algorithm. FCM is an unsupervised statistical classification method which groups the voxels in a given image into different clusters. This algorithm is based on the idea that voxels within a cluster exhibit more similarity when compared the voxels between the clusters. FCM is a soft clustering approach unlike K-means where every image voxel is assigned a certain degree of membership value to the clusters centre points. The membership value provides information to what the extent the image voxels belong to an individual cluster centre. FCM is an iterative algorithm which continuously updates the cluster centre and membership value based on the cost function given in Eq. (1):

1 $$J = \sum\limits_{J = 1}^{N} {\sum\limits_{i = 1}^{C} {w_{ij}^{m} } } \left\| {x_{j} - c_{i} } \right\|^{2}$$

where ${w}_{ij}^{m}$ $w_{ij}^{m}$ variable represents the membership of *x*_*j*_ ${x}_{j}$ data point for the *i*th cluster, *c*_*i*_ is the centroid point value of a cluster centre and the parameter m decides the fuzziness of the partition. Image voxels are segregated into different clusters based on the similarities in their intensity values. The cluster centroids and the membership value of data points were updated iteratively using the Eqs. (2 and 3) respectively.

2 $$C_{i} = \frac{{\sum\nolimits_{J = 1}^{N} {W_{ij}^{m} x_{j} } }}{{\sum\nolimits_{J = 1}^{N} {W_{ij}^{m} } }}$$

3 $$W_{ij} = \sum\limits_{k = 1}^{c} {\left( {\frac{{\left\| {x_{j} - c_{i} } \right\|}}{{\left\| {x_{j} - c_{k} } \right\|}}} \right)}^{2}$$

Although the conventional FCM algorithm outperforms many traditional techniques (for example local thresholding, region growing, KNN and others), we observed that the results were suboptimal when the input images were corrupted by noise and suffers from intensity inhomogeneity effects [19], 31]. In this study we have adopted the FCM code developed by Guanglei Xiong (https://in.mathworks.com/matlabcentral/fileexchange/8351-fuzzy-c-means-thresholding?s_tid=prof_contriblnk). It was modified to process the nifti images and segment the brain tumor regions from BRATS 2018 dataset and our clinical dataset. Briefly the algorithm comprises of the following steps in MATLAB 2018: (a) the bias corrected and brain extracted images were read into MATLAB using ‘niftiread’ command. (b) The images were then rotated to vertical axis. (c) to circumvent the inclusion of unwanted voxels in the segmented tumor an inbuilt MATLAB function ‘bwareaopen’ was used to segment the brain tumor regions. (d) Finally, the segmented tumor regions were compared with the ground truth data to get the DSC values, volume measures and the Hausdorff distance measurement values.

### Deep convolutional neural network

The third algorithm whose performance was evaluated using our clinical dataset was the state-of-art multiscale 3D CNN deep neural network with a fully connected conditional random (CRF) field developed by Kamnitsas et al. [32]. Several 2D CNN models were proposed earlier for different biomedical image segmentation applications [33–36]. However, processing a 3D MRI volume slice by slice using these algorithms is not optimal. Kamnitsas et al.’s algorithm was designed to process 3D volumetric data using parallel 3D convolutional pathways along with a post-processing CRF step to refine the final segmented output image. In brief, the algorithm comprises of twofold pathway architecture with 11 layers in each path. Although the parallel pathways share similar number of deep layers, their functionalities differ with each pathway performing a specific task. The second pathway was designed to capture global spatial information from the down sampled images whereas, the first one processes local information from the 3D patches of multimodal MRI. In both the parallel pathways the network uses small 3^3^ kernels to enable faster convolution with the 3D input patches and to minimize the number of trainable parameters. Once the soft segmented feature maps were extracted from the convolutional layers, they were fed into fully connected CRF to remove the false positive predictions and classify the tumor into edema, active, necrotic and whole tumor regions respectively.

We have taken the Python code from Kamnitsas K GitHub open source repository and studied its performance on our clinical dataset. Apparently, the deepmedic code which is available online cannot be applied directly on our clinical dataset because it was developed to process the 3D MRI images from BRATS dataset. In addition, the deepmedic network trained with the BRATS dataset can only be tested on the images with similar parameters as that of the training dataset (i.e. scanner type, image dimensions, the image acquisition protocol used and others). Therefore, it was modified (the parameter settings) to suit our clinical dataset (which has 2D low resolution FLAIR images and 3D high resolution T1-Gd images) for brain tumor segmentation. The steps include: (a) skull stripped images as input. (b) In order to train and test the deepmedic network, all input images should have same matrix dimension. An affine transformation (12 parameter model) was applied to the 3D T1w-Gd image to down sample and to match its dimensions (the size of 3D T1w-gadolinium contrast enhanced image (image dimension: 512 × 464 × 160) to the reference image i.e. the 2D FLAIR image sequence (image dimension: 512 × 448 × 20). FLIRT linear registration tool from FSL was used for the affine transformation. (d) The training dataset was fed into the deepmedic pipeline and specified the configuration file. (e) The size of image dimension was altered from the default 37 × 37 × 37 to 18 × 18 × 18 in the model configuration file so that the specified dimensions fairly match with the input image size. (f) Then the size of the inference image patch was also customized to 19 × 19 × 19. In addition to these changes, we have varied the number of epochs and sub-epochs from the default 35 to 70 and 20 to 40 respectively. We found that the mean accuracy rate, specificity and sensitivity values reached an optimum at 35th epoch and did not show much variation in values when epoch number was increased beyond 35. (g) Similarly for our clinical dataset all the above network parameter steps were customized.

We trained and tested the deepmedic network with BRATS 2018 dataset and clinical dataset separately. From the BRATS 2018 dataset, 48 randomly chosen subjects (both HGG n = 24 and LGG n = 24 patients) T1-Gd and FLAIR image sequences were used to train the network. We choose 48 subjects from BRATS dataset to closely resemble the sample size of our clinical dataset and we considered equal sample sizes from LGG (n = 24) and HGG (n = 24). In the testing phase, a different set of 10 LGG and 10 HGG patients’ was used to assess trained network’s performance. For the clinical dataset we used 10 HGG and 20 LGG patients’ data to train the network. The trained network’s performance was tested using 8 LGG and 5 HGG cases from our clinical data. DSC values, volume measure and Hausdorff distance measurement was used to analyse the performance of the deepmedic network by comparing it with FCM and RG algorithms on the same dataset.

## Results

### Performance of the tumor segmentation algorithms evaluated using DSC measure

#### Deepmedic algorithm for tumor segmentation

The average performance of deepmedic network on training data (HGG, n = 24 and LGG, n = 24 patients) from BRATS 2018 database is given in Table 1 below.

**Table 1:** Show training metric average DSC score for all the 48 BRATS subjects for different tumor components

Method/algorithm	Entire tumor + edema	DSC Edema	Necrotic
Deepmedic	0.975	0.921	0.911

#### Performance of tumor segmentation algorithms on BRATS 2018 LGG and HGG test dataset

We tested the above trained deepmedic network by using randomly sampled 10 LGG and 10 HGG cases from the BRATS 2018 database. The performance of all the three different brain tumor segmentation algorithms considered in this study was evaluated with this same test data by considering the different tumor components individually. The DSC scores are given in Table 2 below. It can be seen from this table that the DSC score of deepmedic algorithm has dropped significantly to a low value when compared to the DSC scores on the training data as given in Table 1.

**Table 2:** Average DSC score on a different LGG and HGG testing dataset from BRATS 2018 database

METHOD	*DSC (LGG)* Tumor + edema	Edema	Necrotic	*DSC (HGG)* Tumor + edema	Edema	Necrotic
Deepmedic	0.59	0.46	0.36	0.63	0.49	0.39
FCM	0.58	0.47	0.39	0.67	0.55	0.41
Region growing	0.54	–	–	0.63	–	–

Performance of the FCM and region growing algorithms were similar to that of the deepmedic algorithm.

#### Deepmedic network trained using our clinical MRI data

Initially we provided few test sample MRI data from our clinical dataset to the deepmedic network trained using BRATS 2018 dataset for tumor segmentation. But, due to the differences in image matrix dimensions, anisotropic voxels as opposed to isotropic and high resolution images considered in the trained deepmedic network, it could not process the images (was not running giving error). Therefore, we up-sampled the clinical images to match the image dimensions with BRATS dataset using FSL software and tested the resampled images with the network. The results were very poor and the dice score was less than 0.35. Therefore, we trained the deepmedic network using samples of our clinical dataset. The performance of the deepmedic network evaluated using DSC score on the training dataset is given in Table 3 below.

**Table 3:** Show training metric average DSC score from deepmedic algorithm for our clinical dataset

Method	Entire tumor + edema	DSC Edema	Necrotic
*Deepmedic*	0.978	0.941	0.933

#### Performance of the tumor segmentation algorithms on our clinical test LGG and HGG dataset

**Fig. 1:**
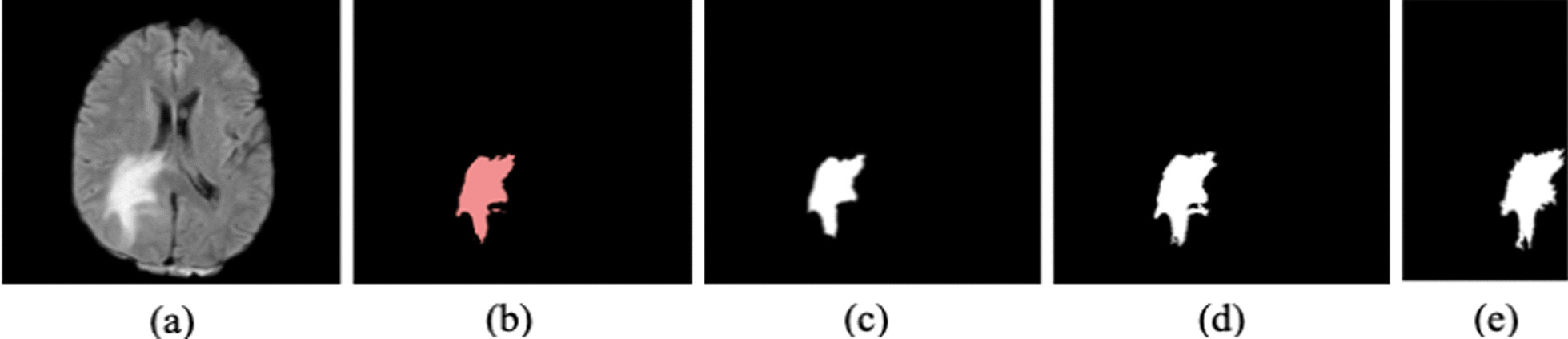
Results from the three algorithms applied on a typical LGG patient from our clinical dataset. **a** FLAIR image sequence, **b** ground truth image, **c** segmented tumor region from deepmedic algorithm, **d** segmented tumor region from FCM, **e** segmented tumor region from region growing algorithm

**Fig. 2:**
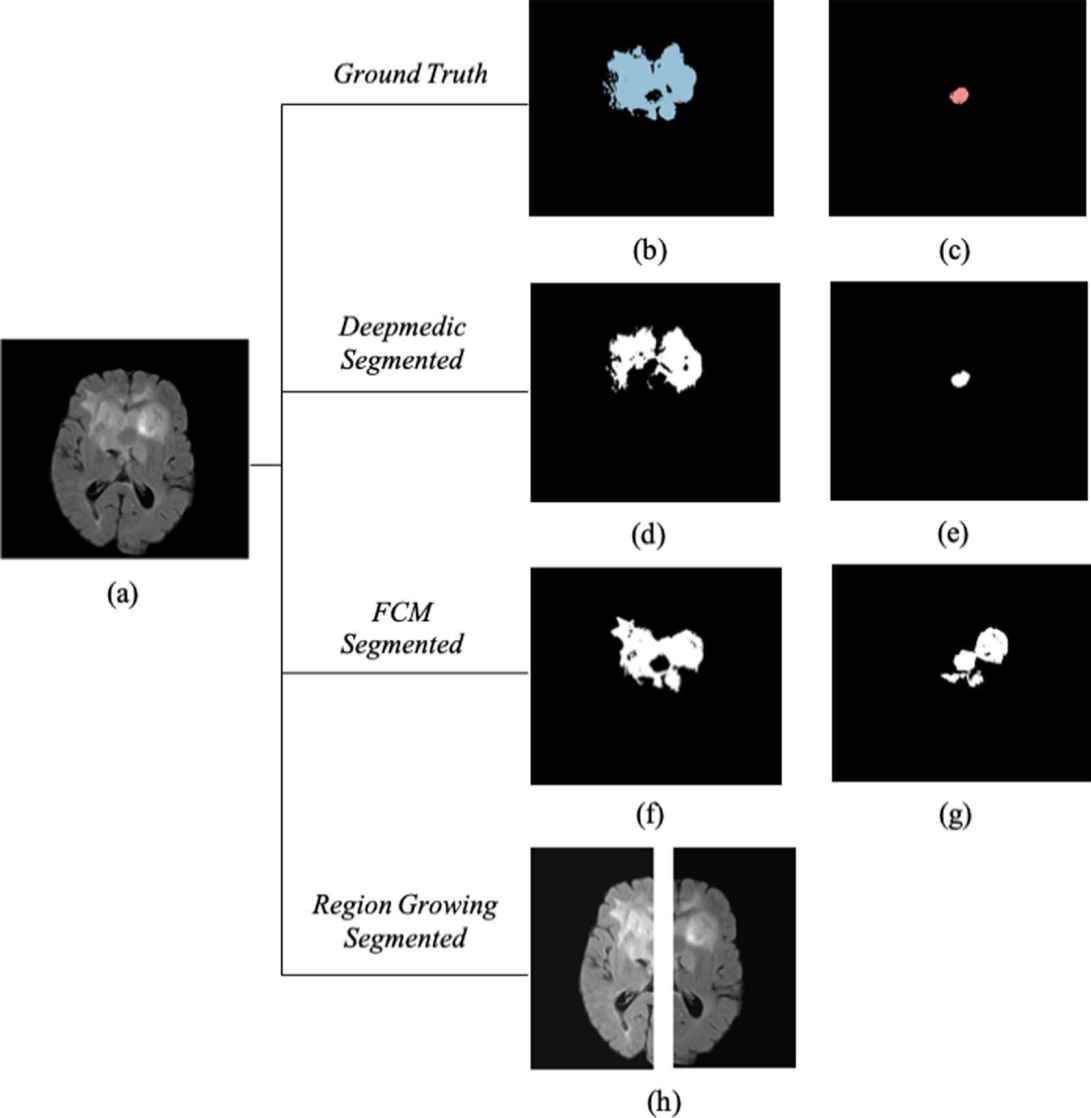
The three algorithms were applied on a typical patient from our clinical dataset. **a** FLAIR image sequence, **b** segmented ground truth of edema region using ITK snap tool, **c** segmented ground truth of necrotic region using ITK snap tool, **d** segmented edema region using deepmedic algorithm, **e** segmented necrotic region using deepmedic algorithm, **f** segmented edema region using FCM, **g** segmented necrotic region using FCM, **h** In the image we tried to use the half brain symmetrical property to segment the brain tumor region. Since, the brain tumor is spread into both the hemispheres our assumption in the development of region growing algorithm is violated. So, region growing algorithm fails to segment the tumor in such type of images

Deepmedic network which was trained using our clinical dataset is tested using a different set of randomly chosen LGG and HGG testing data from our clinical database. To evaluate the performance of the deepmedic network we used 8 LGG and 5 HGG patients. For comparison purposes we have also segmented the tumor in these 13 patients (8 LGG and 5 HGG) using RG and FCM algorithms. These results are given in Table 4 below. Figures 1 and 2 shows the typical LGG and HGG tumor segmentation results from all the three algorithms along with the ground truth segmented images.

**Table 4:** Average DSC scores on our clinical testing dataset

METHOD	*DSC (LGG)* Tumor + edema	Edema	Necrotic	*DSC (HGG)* Tumor + edema	Edema	Necrotic
Deepmedic	0.80	0.67	0.45	0.66	0.54	0.40
FCM	0.55	0.46	0.38	0.634	0.56	0.39
Region growing	0.46	–	–	0.58	–	–

The performance of the deepmedic algorithm was comparatively better than the FCM and the RG algorithms for all the tumor components. In LGG tumor patients deepmedic showed superior performance especially for the entire tumor + edema component when compared to HGG tumor patients.

### Performance of the tumor segmentation algorithms evaluated using Hausdorff measurement

In addition to the DSC score Hausdorff distance measure was also measured to evaluate the performance of the segmentation algorithms in accurately detecting/identifying the boundary of the tumor and its components.

#### Performance of the tumor segmentation algorithms on BRATS 2018 LGG and HGG test dataset

Hausdorff distance values are computed by measuring the distance between the boundary of the segmented tumor and its components with the ground truth data. Hausdorff distance will be low when there is a perfect match between the segmented region boundary with its corresponding ground truth region boundary and vice versa. Table 5 below gives Hausdorff distance values for the LGG and HGG BRATS test data.

**Table 5:** Average Hausdorff distance values for LGG and HGG BRATS test 2018 dataset

METHOD	*LGG Hausdorff (voxels)* Tumor + edema	Edema	Necrotic	*HGG Hausdorff (voxels)* Tumor + edema	Edema	Necrotic
Deepmedic	13.43	20.23	22.40	11.59	14.68	12.86
FCM	27.27	25.34	32.80	18.23	20.26	25.08
Region growing	23.46	–	–	21.00	–	–

When compared to FCM and RG approach deepmedic gave better results (Hausdorff values are lower) i.e. the tumor and its component boundaries closely matched with the ground truth data.

#### Performance of the tumor segmentation algorithms on our clinical test LGG and HGG dataset

Hausdorff measurement results for different tumor segmentation algorithms for our clinical LGG and HGG data set is given in Table 6.

**Table 6:** Average Hausdorff dimension values for our clinical LGG test dataset

METHOD	*LGG Hausdorff (voxels)* Tumor + edema	Edema	Necrotic	*HGG Hausdorff (voxels)* Tumor + edema	Edema	Necrotic
Deepmedic	7.47	13.05	11.40	10.65	12.91	14.75
FCM	15.81	14.73	18.20	16.50	20.23	21.30
Region growing	31.33	–	–	32.27	–	–

Similar to BRATS dataset results, deepmedic gave better results on the clinical dataset (Hausdorff dimension values are lower) when compared to FCM and region growing approach. These results show that deepmedic performance is consistent across BRATS and clinical data set.

### Performance of the tumor segmentation algorithms evaluated using volume measure

Tumor volume is an important measure used in clinical and radiological practice to understand the growth rate of benign and malignant tumor regions. Therefore, we also evaluated the performance of the three tumor segmentation algorithms by measuring the volume of the segmented tumor and its components and comparing them with the ground truth data.

#### BRATS 2018 LGG and HGG dataset

Volume measure is basically the total number of voxels in a given segmented region. Volume of the segmented tumor and its components given by deepmedic, FCM and RG algorithms for LGG and HGG subjects from BRATS 2018 dataset is given in Table 7 below along with the volumes measured from ground truth data.

**Table 7:** Volume of the segmented tumor and its components by deepmedic, FCM and region growing algorithms for LGG and HGG BRATS 2018 dataset

METHOD	LGG						HGG					
	Edema + tumor (GT)	Edema + tumor	Edema (GT)	Edema	Necrotic (GT)	Necrotic	Edema + tumor (GT)	Edema + tumor	Edema (GT)	Edema	Necrotic (GT)	Necrotic
Deep-medic	0.17 M	0.12 M	0.10 M	0.08 M	0.06 M	0.05 M	0.19 M	0.15 M	0.10 M	0.07 M	0.01 M	0.008 M
FCM	0.17 M	0.24 M	0.10 M	0.18 M	0.06 M	0.08 M	0.19 M	0.16 M	0.10 M	0.12 M	0.01 M	0.03 M
RG	0.17 M	0.11 M	–	–	–	–	0.19 M	0.12 M	–	–	–	–

*GT* ground truth, *RG* region growing

In general the volume of the tumor and its components measured from the segmentation results obtained using deepmedic algorithm was reduced (underestimated) when compared to the ground truth tumor volume and its components. On the other hand the volume of the tumor and its components from FCM algorithm was higher (overestimated) when compared to the ground truth. The volume of the tumor and its components given by the RG algorithm was very lower (much underestimated when compared to deepmedic).

#### Our clinical dataset

The volume of the tumor and its components measured using our clinical data to understand the performance of the three tumor segmentation algorithms is given in Table 8.

**Table 8:** Volume of the segmented tumor and its components by deepmedic, FCM and region growing algorithms for the clinical dataset

METHOD	LGG						HGG					
	Edema + tumor (GT)	Edema + tumor	Edema (GT)	Edema	Necrotic (GT)	Necrotic	Edema + tumor (GT)	Edema + tumor	Edema (GT)	Edema	Necrotic (GT)	Necrotic
Deep-medic	0.13 M	0.14 M	0.08 M	0.08 M	0.06 M	0.08 M	0.33 M	0.28 M	0.26 M	0.21 M	0.03 M	0.02 M
FCM	0.13 M	0.16 M	0.08 M	0.13 M	0.06 M	0.03 M	0.33 M	0.25 M	0.26 M	0.2 M	0.03 M	0.06 M
RG	0.13 M	0.09 M	–	–	–	–	0.33 M	0.23 M	–	–	–	–

## Discussion

The main findings of this study are the following (1) results show that even the sophisticated deep neural networks like ‘deepmedic’ cannot be used without re- training on the ‘custom dataset’. (2) It is expected that even the sophisticated deep neural networks like ‘deepmedic’ when trained on small sample size training dataset performs poorly on the ‘test dataset’. This suggests that even if one wants to customize an available robust neural network architecture like deepmedic for their custom ‘clinical dataset’ the results/performance are far from what is expected in the clinical domain. Deep neural networks require large training data in order achieve superior performance (for e.g. to achieve DSC score of above 0.8). (3) Among the three segmentation algorithms RG performed poorly, FCM showed better performance when compared to RG but is suboptimal when compared to the deep neural networks.

The primary reasons for choosing the deepmedic architecture for this study was the availability (open source) of its validated python code. The network has a robust design with two pathway architecture to handle local and global information for the brain tumor segmentation problem. The fully connected CRF minimizes false positive predictions and moreover the deepmedic network was ranked 1st in BRATS 2016 challenge with the DSC scores of 0.901, 0.754, and 0.728 for whole tumor, tumor core and enhanced tumor regions respectively. We trained the deepmedic network using 48 subjects (LGG + HGG) from BRATS 2018 database and employed the network to segment our clinical data by up-sampling the clinical images (i.e. to match the image dimensions with BRATS dataset). The network performed poorly on our clinical images with a DSC score below 0.7 (usually a network’s performance is said to be good when its DSC score is above 0.7 [37]). Our results show that the network performed poorly when a different test dataset from the BRATS database was given for segmentation. Inter subject variations in DSC scores were observed in our data i.e. in few patients we have observed a very low DSC scores which in turn may affect the average DSC scores presented in the results section. Being a preliminary study with small sample size we did not perform any outlier rejection procedure while calculating the average values. The consistent poor performance of the network in both the BRATS and on our clinical test dataset indicate that differences in imaging parameters between the BRATS dataset and our clinical dataset did not affect the network performance.

Comparing Tables 1 and 2, deepmedic goes from extremely high training metrics to low testing metrics. We believe that this is probably due to over-fitting of the model and due to the small sampled size training dataset. Even though the exact reason why deepmedic showed slightly better performance on our clinical dataset when compared to the high resolution BRATS test dataset is not clear to us at this time point. One probable reason is in our clinical dataset the location of the tumor in brain hemispheres is similar in both the test and training dataset which may not be the case with the BRATS dataset (since we picked the images randomly from BRATS dataset).

The network also did not show any significant difference in its performance between the LGG and HGG tumor patients. On the other hand the network revealed superior performance in segmenting the same training dataset. The above results clearly demonstrate that DNN requires large training data with diversity in it. A large diverse training dataset will enable the network to learn in a robust way. This will result in superior segmentation results for the unseen/ test data. We believe that the results seen here with our clinical data will improve significantly if more number of training dataset can be included while training the network. We took only 48 cases from BRATS dataset (when we trained the network on the BRATS dataset) in order to resemble the sample size of our clinical dataset (which is 43). This was done to understand the difference between training a network using high resolution isotropic images as opposed to anisotropic low resolution clinical images by excluding the effect of sample size. However, our conclusion to use ‘larger datasets needed for deep learning’ comes from the results seen on the validation dataset from both these networks i.e. the network trained using BRATS dataset and the network trained using our clinical dataset. In both these results validation performance decreased well below from what was claimed by the authors [Kamnistats et al. [32] when they trained the network using large dataset (220 patient images).

A recent study [38] which employed the latest state of the art ‘densenet’ type of architecture also showed DSC values less than 0.8 when their clinical data was given as input for tumor segmentation on the pre-trained network using BRATS dataset. We also tried using the U-net architecture for tumor segmentation from the opensource github repository (https://github.com/IAmSuyogJadhav/Brainy). When our clinical data was given as input to the pre-trained U-Net architecture, it failed to segment the tumor regions. The U-net instead segmented the non-tumorous brain region pixels as clusters. Hence, we could not measure the efficacy of the U-Net architecture. These results clearly demonstrate/emphasize the need to design a robust deep neural architecture which can be readily applied to a clinical dataset with ease.

Lower Hausdorff measurement values obtained for the tumor and its components in both BRATS dataset and on our clinical dataset clearly indicate that deepmedic is able to accurately detect/segment the boundary of the tumor and its components when compared to FCM and the RG algorithms. The results from volume measure also supports the view that deepmedic may be a better segmentation algorithm when compared to FCM and RG algorithms. Both FCM and RG overestimates the volume of the tumor and its components whereas, deepmedic underestimates it. Even though underestimation of tumor and its components volume is unacceptable in clinical practice we believe that when trained using large diverse dataset, deepmedic may overcome this drawback. During the training phase of the network we also varied the network training parameters like the number of epochs to study their effect on the tumor segmentation accuracy. As the epoch number was reduced from the default settings, a reduction in the DSC values was observed.

Even though the DSC scores of FCM and deepmedic algorithms were close to each other for BRATS and clinical dataset in this study we believe that deepmedic algorithm has the potential to perform better because in deepmedic network multimodal MR sequences were used to train the network which is may be more robust for tumor segmentation. The multimodal MR sequences such as FLAIR, T1-Gd and T1-w will definitely provide more information to achieve nearly precise segmentation results. In the case of FCM each slice is processed individually unlike the deepmedic algorithm where the entire 3D data is processed. One limitation of deepmedic over the FCM is the time and computing resources required to train the network. Since, a deep learning model trains, optimizes and fine-tune billions of parameters, our experience shows that it requires a high end workstation of at least 16 GB of RAM and a high end GPU processor to perform fast calculation. In this study, we trained the deepmedic network on a i7 processor, 8 GB RAM and 4 GB NVIDIA GTX 1650 GPU. It took nearly 96 h to train the given input images.

The performance of the RG algorithm was very poor when compared to FCM and deepmedic in terms of Hausdorff measurement values and volume measures. The main constraint with our RG algorithm was that it assumes brain half symmetry and the tumor is present in one of the hemispheres only. Therefore, it gives superior performance only in cases where the tumor is present in one of the hemispheres but not in both. For most of our patients in the clinical dataset the above assumptions was violated as the tumor was present asymmetrically in both the brain hemispheres. Further, like FCM our RG algorithm accepts only a single modality input image sequence. Our previous studies [27] showed that our RG algorithm works well when using T2-w data set when compared to FLAIR images. Also, the input images to the RG algorithm should be in the form of individual MRI 2D tumor slices as opposed 3D volumetric dataset considered in the deepmedic network. In addition to the above, the main limitation of our RG algorithm is that it cannot output the segmented tumor and its components individually like deepmedic algorithm. Comparison between FCM and our RG algorithm shows that FCM performs better than RG algorithm in detecting the boundary of the tumor and its components.

When considering the results of the segmented tumor and its components, we could see from DSC, Hausdorff and volume measures all the three tumor segmentation algorithms gave good accuracy (in terms of DSC, volume, Hausdorff dimension of the boundary) for the tumor + edema region (that is the entire tumor and its constituent region) when compared to either the edema or necrotic region considered alone. Even though the reason for this discrepancy in the performance of the algorithms is unclear to us at the moment, a robust tumor segmentation algorithm should not only identify/segment the entire tumor and its components accurately but also the individual components of the tumor i.e. necrotic region, active tumor region and edema region.

## Conclusion

In this study we aimed to identify a robust segmentation algorithm for clinical dataset with low resolution and non-contiguous slices. We compared segmentation algorithms at different levels of the hierarchy. This was done for the following reason i.e. if the segmentation algorithms in lower levels of hierarchy show good performance, then computationally expensive DNN algorithms can be avoided for routine clinical use. DNN algorithms may be better suited for such kind of clinical data because DNN can use 3D information from 3D dataset and can provide individually the volumes of the segmented tumor and its constituents. DNN algorithms require large diverse training dataset and require high computational power. RG algorithm with brain half symmetry cannot be used to segment brain tumors in patients where the tumor is present in both the brain hemispheres asymmetrically. FCM algorithm is robust when compared to RG but fails to consider multimodal input images which provide different tissue contrast information of the tumor and its constituents.

## Data Availability

The datasets generated and/or analysed during the current study are not publicly available as it is the proprietary property of SCTIMST. Any queries related to this can be directed to the corresponding author, Venkateswaran Rajagopalan at the Email: venkateswaran@hyderabad.bits-pilani.ac.in. BRATS 2018 dataset is available at https://www.med.upenn.edu/sbia/brats2018/data.html.
